# Updated cost-of-care estimates for commercially insured patients with multiple sclerosis: retrospective observational analysis of medical and pharmacy claims data

**DOI:** 10.1186/1472-6963-14-286

**Published:** 2014-07-02

**Authors:** Cathryn A Carroll, Kathleen A Fairman, Maureen J Lage

**Affiliations:** 1 Managed Markets, Xcenda; formerly Health Economics and Outcomes Research, Teva Pharmaceuticals, Kansas City, MO, USA; 2 Kathleen Fairman LTD, 11208 North 23rd Place, Phoenix, AZ, USA; 3 HealthMetrics Outcomes Research, LLC, Bonita Springs, FL, USA

## Abstract

**Background:**

For patients with multiple sclerosis (MS), previous research identified key disease sequelae as important cost drivers and suggested that among users of disease-modifying drugs (DMDs) in 2004, DMDs represented 73% of the total cost of care. More recent studies were limited to incident disease/treatment and/or excluded DMDs from cost estimates. To support contemporary pharmacoeconomic analyses, the present study was conducted to provide updated information about MS-related costs and cost drivers including DMDs.

**Methods:**

For each of 2 years, 2006 and 2011, commercially insured, continuously eligible patients with ≥ 1 medical claim diagnosis of MS were sampled. MS-related charges were based on medical claims with MS diagnosis plus medical/pharmacy claims for DMDs. 2006 charges were adjusted to 2011 $ using the medical care component of the consumer price index (CPI). Subgroups of patients using DMDs (interferon [IFN] beta-1a intramuscular or subcutaneous, IFN beta-1b, glatiramer, natalizumab) in 2011 were identified. By-group differences were tested with bivariate statistics.

**Results:**

Mean (standard deviation [SD]) age of 15,902 sample patients in 2011 was 47.6 (11.8) years, 76% female. Mean [SD] MS charges ($26,520 [$38,478] overall) were significantly (*P* < 0.001) higher for patients with common disease sequelae: malaise/fatigue (n = 2,235; $39,948 [$48,435]), paresthesia (n = 1,566; $33,648 [$45,273]), depression (n = 1,255; $42,831 [$51,693]), and abnormality of gait (n = 1,196; $48,361 [$55,472]). From 2006 to 2011, CPI-adjusted MS charges increased by 60%. Among patients treated with a single DMD in 2011, inpatient care was 6% of charges (range = 4%-8%; *P =* 0.155); outpatient care was 19% (range = 14%-20% except for natalizumab [29%]; *P* < 0.001); and DMDs were 75% (range = 67%-81%; *P* < 0.001).

**Conclusions:**

Common MS sequelae remain important cost drivers. Although MS treatment costs are increasing, the proportion of MS charges due to DMDs in 2011 is similar to that reported in 2004.

## Background

Multiple sclerosis (MS) is a chronic, disabling disorder of the central nervous system that typically presents initially in early adulthood, with symptoms and disease progression rates that vary widely among patients [1]. Although MS is rare, affecting less than 0.1% of the U.S. population [2], its effects on patient functioning and on medical service utilization result in high direct and indirect costs, estimated in 1998 to total $2.2 million per U.S. case per lifetime [3]. Natural history studies conducted prior to the introduction of disease-modifying drugs (DMDs) to the market (initially interferon [IFN] beta-1b in 1993; IFN beta-1a intramuscular [IM] and glatiramer acetate in 1996) [4] found considerable functional deterioration in the years following disease onset [5]. For example, at 10 years post-onset, one-half of patients were unable to perform daily employment or household activities; and at 15 years, one-half were unable to walk without assistance [5].

DMD treatment of MS early in the disease course, especially for patients with relapsing-remitting MS (RRMS) and to a lesser extent for patients at risk of developing clinically definite MS (CDMS), is becoming a standard of care in the United States [4,6]. In 2002, a joint task force of the American Academy of Neurology and MS Council for Clinical Practice Guidelines recommended that IFN beta treatment be considered “in any patient who is at high risk for developing [CDMS], or who already has either RRMS or secondary progressive MS (SPMS) and is still experiencing relapses” and that treatment with glatiramer be considered for patients with RRMS [6]. A 2008 disease management consensus statement from the National Multiple Sclerosis Society indicated that treatment with an IFN beta medication or glatiramer “should be considered as soon as possible following a definite diagnosis of MS with active, relapsing disease, and may also be considered for selected patients with a first attack who are at high risk of MS” [7].

Growing consensus about the use of DMDs in MS treatment has heightened the importance of MS cost-of-care analyses for health care payers, including employers. DMDs are high-cost medications, with one economic analysis estimating total treatment expense including laboratory monitoring costs at $2,294 to $2,461 per month (2008 U.S.$) [8]. However, because both direct medical care costs and societal costs (e.g., payments for disability and sick leave, value of caregiver time) increase as the disease advances [9], the efficacy of DMDs in slowing the progression of MS has the potential to offset drug acquisition costs with savings on other resources.

For example, Prescott et al. found that among patients using a single DMD (monotherapy) in 2004, 75% of total MS treatment cost was attributable to prescription drugs, with 73% solely for the DMD [10]. However, they compared their results with those of a previous cost-of-care estimate by Pope et al. and concluded that after accounting for inflation, “the cost structure for treating MS has changed notably”—from a total 1-year cost of $9,515 with 18% for prescription drugs in 1995, to $12,879 with 65% for prescription drugs in 2004 [10,11]. Estimating that inflation-adjusted annual medical cost per patient with MS had declined from $7,802 in 1995 to $4,529 in 2004, Prescott et al. suggested that the downward shift “may be related to the effectiveness of DMD therapy in managing MS severity and reducing functional disability” [10].

In addressing questions about effective treatment approaches and cost of care, payers commonly turn to published observational studies or to pharmacoeconomic models, which rely on input data to produce information about expected “real-world” costs and outcomes [12,13]. However, published data about MS treatment costs are not timely or complete. Early cost estimates by Whetten-Goldstein et al. and Pope et al. were based on data collected in 1994–1997 [3,11]. The study by Prescott et al. provided a comprehensive descriptive analysis of the components of MS treatment costs for a large sample but was based on dates of service in 2004 [10]. Analyses published since then have been subject to several common limitations. Many studied only patients with incident disease and/or treatment, who might not accurately represent payers’ cost experiences with prevalent disease populations [14-19]. All studied patients initiating treatment prior to 2009 [14-20]. Several excluded DMD costs in reporting the cost of care [16,18,20]. These inadequacies in the evidence base are consequential because of important changes in MS treatment in recent years: the publication of several new treatment guidelines [21,22]; the withdrawal of natalizumab from the market in February 2005 followed by its reintroduction in June 2006 under a risk mitigation strategy [23,24]; the approval of a new, branded IFN beta-1b in August 2009 [25]; and the availability of an oral DMD, fingolimod, beginning in September 2010 [4].

To address these gaps in the research literature, the primary objective of the present study was to support pharmacoeconomic analyses of MS treatments by providing an updated assessment of direct health care costs in a contemporary, prevalent cohort of patients with MS, replicating the work of Prescott et al. as closely as possible [10]. The secondary objective, again replicating the method used by Prescott et al., was to assess the growth in medical and drug costs over time by comparing aggregated cost data for prevalent samples in 2006 and 2011.

## Methods

### Design and data source

The study was a retrospective analysis of the i3 Invision™ Data Mart, an integrated database of medical claims, pharmacy claims, and eligibility data for approximately 14 million enrollees of a national health insurance organization. All enrollees were commercially insured, and most were located in the southern or midwestern United States. Available fields on medical claims included *International Classification of Diseases, Ninth Revision, Clinical Modification* (ICD-9-CM) codes for up to 5 diagnoses, as well as procedure codes (Current Procedural Terminology [CPT], revenue, and Healthcare Common Procedure Coding System [HCPCS]). Pharmacy claims fields included drug brand names, national drug code (NDC) numbers, fill dates, and days supply. Data were collected as part of normal business operations and de-identified prior to delivery to the investigators in accordance with Health Insurance Portability and Accountability Act (HIPAA) requirements. Although the study by Prescott et al. used proprietary Episode Treatment Group (ETG) software to calculate some study outcomes [10], the present study used only data elements that are widely available in administrative claims databases.

### Sample selection

The same sampling process was used to produce MS patient cohorts for each of the 2 study years, 2006 and 2011. All study patients had (1) at least 1 medical claim with a diagnosis of MS, defined as either an ICD-9-CM = 340.xx in any diagnosis field or a diagnosis-related group (DRG) code for MS and cerebellar ataxia (DRG = 058, 059, or 060) at any time during the study year *and* (2) continuous eligibility for medical and pharmacy benefits throughout the study year.

For 2011, a subgroup of patients with at least 1 claim for a DMD was created. From this subgroup, an additional subgroup of patients treated with claims for only a single DMD (monotherapy) was identified. DMDs included IFN beta-1a subcutaneous (SC), IFN beta-1a IM, IFN beta-1b, glatiramer acetate, and natalizumab and were identified either by brand name on pharmacy claims or HCPCS codes on medical claims (Table 1).

**Table 1:** Disease-modifying drugs for multiple sclerosis

**Brand Name**	**Generic Name**	**HCPCS Codes**^**a**^
Avonex^b^	IFN beta-1a IM^b^	J1825, Q3025
Betaseron^b^, Extavia	IFN beta-1b SC^b^	J1830
Copaxone^b^	Glatiramer acetate^b^	J1595
Gilenya	Fingolimod	None
Rebif^b^	IFN beta-1a SC^b^	J1826, Q3026
Tysabri	Natalizumab	J2323, Q4079

^a^Drugs were identified by brand names on pharmacy claims and HCPCS codes on medical claims.

^b^Indicates a DMD included in the study by Prescott et al. [10].

DMD = disease-modifying drug; HCPCS = Healthcare Common Procedure Coding System; IFN = interferon; IM = intramuscular; SC = subcutaneous.

### Analysis

Consistent with the analyses performed by Prescott et al., study outcomes reported for 2011 included mean (standard deviation [SD]) charges for MS services by geographic region, insurance type, age group, sex, and presence of selected disease sequelae, which were described as comorbidities in the report by Prescott et al. MS services were defined based on a diagnosis of MS (ICD-9-CM or DRG as described above) in any field on medical claims or receipt of a DMD as indicated in either medical or pharmacy claims. Costs were defined as submitted charges minus any expense that was not covered because of incomplete information or ineligible services, patients, or providers. For this reason, we use the term “charge” rather than “cost” throughout the rest of this report.

To identify disease sequelae, diagnoses in any field were used. Each condition (abnormality of gait; ataxia; paresthesia [burning, numbness, or tingling]; convulsions; depression; fecal incontinence; fibromyalgia/myalgia; malaise and fatigue; optic neuritis; spasms; trigeminal neuralgia; urinary incontinence; and voice disturbance) was identified using coding similar to that of Prescott et al., except that: (1) Prescott et al. used a combination of ICD-9-CM codes and ETGs to identify disease sequelae, and (2) in the present study, a DRG of 881 (depressive neuroses) was used in addition to ICD-9-CM coding to identify depression.

Additional analyses for 2011 included assessments of MS-related utilization and charges by service category (inpatient, outpatient emergency room, and DMD) and separate analyses of DMD monotherapy users by drug. Although patients treated with fingolimod were included in the sample overall, they were excluded from the DMD-specific analyses because of small subgroup size (n = 306). A final analysis compared the distribution of MS-related total, medical, and DMD charges for the 2 years, 2006 and 2011, with 2006 charges adjusted to 2011 values using the medical care component of the Consumer Price Index.

Although the analytic approach was descriptive, inferential statistical tests, including Pearson chi-square for categorical variables and t-tests and Analysis of Variance (ANOVA) for interval- or ratio-scale variables, were used [10]. No multivariate adjustments were made because the primary purpose of the study was to provide descriptive cost data for input into pharmacoeconomic models, not to assess the cost-effectiveness of the various available DMDs. Statistical analyses were performed using SAS version 9.2 and an *a priori* significance level of 0.05.

## Results

**Figure 1:**
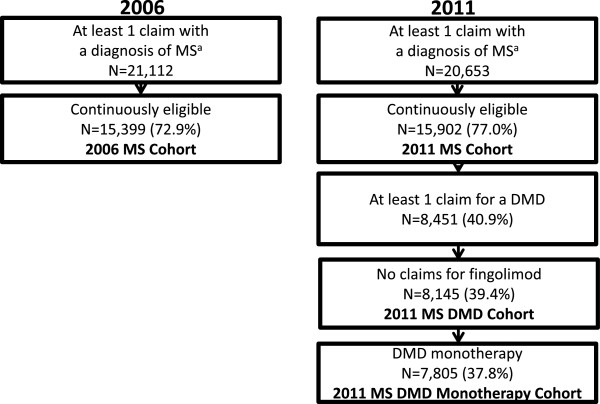
Sample selection process and cohort identification.

Of potential study subjects with an MS diagnosis or DRG code during each of the 2 study years (n = 21,112 in 2006 and 20,653 in 2011), more than 70% (n = 15,399 in 2006 and 15,902 in 2011) were continuously eligible and constituted the MS patient cohort (Figure 1). Of continuously enrolled patients, the proportions with at least 1 DMD claim were 54% (n = 8,248) in 2006 and 53% (n = 8,451) in 2011.

The mean (SD) age for the 2011 cohort was 47.6 (11.8) years, and 76% of study subjects were female (Table 2). The mean (SD) total MS health care charge in 2011 for the sample overall was $26,520 ($38,478). MS charges were slightly but significantly higher for males compared with females. Charges were distributed in an inverted “U” shape by age, with the lowest charges for patients younger than 18 years (< 1% of the sample; mean of $14,142), increasing incrementally to means of $27,430-$27,558 for those aged 36 to 55 years (57% of the sample), and dropping to a mean of $21,167 among those aged 65 years or older (5% of the sample).

**Table 2:** **MS charges**^**a **^**in 2011**

	**N**	**% of Total**	**Mean [SD] charge per patient ($)**	***P*** **Value**
All patients	15,902	100.0	26,520 [38,478]	
By patient characteristics				
Gender				<0.001^b^
Female	12,100	76.1	26,224 [38,716]	
Male	3,801	23.9	27,445 [37,691]	
Unknown	1	0.0	90,704 [NA]	
Age in years				<0.001
17 or younger	126	0.8	14,142 [40,310]	
18 to 25	405	2.6	25,731 [44,296]	
26 to 35	2,009	12.6	26,324 [36,224]	
36 to 45	4,021	25.3	27,430 [36,866]	
46 to 55	5,030	31.6	27,558 [39,653]	
56 to 64	3,499	22.0	25,876 [38,022]	
65 or older	812	5.1	21,167 [42,005]	
Region				
Northeast	1,861	11.7	27,035 [38,558]	0.646
Midwest	4,480	28.2	25,820 [32,754]	
South	6,730	42.3	26,604 [40,196]	
West	2,827	17.8	27,093 [42,457]	
Other	4	0.0	23,745 [28,601]	
Insurance product				0.032
EPO	2,088	13.1	27,933 [42,360]	
HMO	1,474	9.3	24,628 [33,046]	
Indemnity	329	2.1	22,127 [39,181]	
POS	11,336	71.3	26,648 [38,173]	
PPO	641	4.0	26,622 [41,918]	
Other	34	0.2	19,353 [28,310]	
**Charge components**	**Utilization**^**c**^**Mean [SD]**		**Mean [SD] Cost ($)**	**% of Total Cost**
Inpatient^d^	0.48 [2.75]		3,179 [17,860]	12.0
Outpatient^d^	6.05 [8.04]		9,355 [24,523]	35.3
Emergency Room^d^	0.03 [0.39]		33 [522]	0.1
DMD	3.17 [4.87]		13,953 [21,704]	52.6

^a^Sum of the charges for (1) medical claims with an MS diagnosis (ICD-9-CM = 340.xx in any diagnosis field or a DRG code for MS and cerebellar ataxia [DRG = 058, 059, or 060]) *plus* (2) medical or pharmacy claims indicating a DMD (Table 1).

^b^Excluding the patient with unknown gender (i.e., *t*-test for female versus male).

^c^Number of claims. Claims counts for DMDs include both medical and pharmacy claims.

^d^Charges for medical claims with an MS diagnosis (ICD-9-CM = 340.xx in any diagnosis field or a DRG code for MS and cerebellar ataxia [DRG = 058, 059, or 060]).

DMD = disease-modifying drug; DRG = Diagnosis-Related Group; EPO = exclusive provider organization; HMO = health maintenance organization; MS = multiple sclerosis; NA = not applicable; POS = point of service; PPO = preferred provider organization; SD = standard deviation.

Charges did not significantly differ by geographic region but did differ by insurance product type (Table 2). Among common insurance product types (i.e., not in the “other” category), mean MS charges ranged from a low of $22,127 in indemnity plans to a high of $27,933 in exclusive provider organizations.

For the sample overall (including both DMD-treated patients and those without DMD use), inpatient services made up 12% and DMDs 53% of total MS-related charges in 2011 (Table 2). Measured by claims volume, the most commonly used services were outpatient, constituting 62% of claims and 35% of total MS-related charges.

Subgroup analyses of patients with selected disease sequelae indicated that both high- and low-prevalence conditions were associated with substantially increased MS charges (Table 3). Patients with optic neuritis (n = 38) had the highest mean charges ($82,134; *P* = 0.001), followed by those with abnormality of gait (n = 1,196), spasms (n = 412), and ataxia (n = 347), with means of $48,337 to $48,605 (all *P* < 0.001). Urinary incontinence (n = 339) and depression (n = 1,255) were also associated with high MS charges at means of $47,885 and $42,831, respectively (both *P* < 0.001). The highest-prevalence sequela was malaise and fatigue, diagnosed in 14.1% of patients (n = 2,235) and associated with a mean MS charge of $39,948.

**Table 3:** **MS charges**^**a **^**by presence of selected disease sequelae in 2011**

**Condition**	**Diagnosis code**^**b**^	**N**	**Prevalence**	**Mean [SD] charges ($)**	***P*** **Value**
Abnormality of gait	781.2	1,196	7.5	48,361 [55,472]	<0.001
Ataxia	781.3	347	2.2	48,605 [63,100]	<0.001
Burning, numbness, tingling sensations	782.0	1,566	9.8	33,648 [45,273]	<0.001
Convulsions^c^	780.3	0	0.0	---	---
Depression^d^	296.2, 296.3, 300.4, 311; DRG = 881	1,255	7.9	42,831 [51,693]	<0.001
Fecal incontinence	787.6	0	0.0	---	---
Fibromyalgia/myalgia and myositis	729.1	399	2.5	38,319 [63,441]	<0.001
Malaise and fatigue^c^	780.7	2,235	14.1	39,948 [48,435]	<0.001
Optic neuritis	341.0	38	0.2	82,134 [98,886]	0.001
Spasms	781.0	412	2.6	48,337 [52,475]	<0.001
Trigeminal neuralgia	350.1	150	0.9	38,165 [46,506]	0.002
Urinary incontinence^c^	788.3	339	2.1	47,885 [49,226]	<0.001
Voice disturbance^c^	784.4, 784.5	23	0.1	44,973 [57,858]	0.140

^a^Sum of the charges for (1) medical claims with an MS diagnosis (ICD-9-CM = 340.xx in any diagnosis field or a DRG code for MS and cerebellar ataxia [DRG = 058, 059, or 060]) *plus* (2) medical or pharmacy claims indicating a DMD (Table 1).

^b^ICD-9-CM coding except where otherwise specified.

^c^Identified by Prescott et al. using a combination of ICD-9-CM codes and ETG coding [10].

^d^Identified by Prescott et al. using only ETG coding [10].

DMD = disease-modifying drug; DRG = Diagnosis-Related Group; ETG = Episode Treatment Group; ICD-9-CM = International Classification of Diseases, Ninth Revision, Clinical Modification; MS = multiple sclerosis; SD = standard deviation.

For the subgroup of 8,145 patients with at least 1 injectable DMD claim in 2011, the most frequently used product was glatiramer acetate (40%), followed by IFN beta-1a IM (22%), IFN beta-1a SC (21%), IFN beta-1b (11%), and natalizumab (10%; Table 4). Similar DMD use rates were identified in the subgroup of monotherapy-treated patients. However, both the mean claims count and the mean days supply were lower for glatiramer acetate compared with the other DMDs. A *post hoc* follow-up analysis of this finding indicated that the mean number of days until DMD start, defined as the count of calendar days from January 1, 2011, through the first DMD claim date, was greater (*P* < 0.001) for patients treated with glatiramer acetate (76.4 days) compared with other DMDs (41.6 to 54.2 days).

**Table 4:** DMD utilization and patient characteristics: patients with MS in 2011

	**IFN beta-1a IM**		**IFN beta-1b**		**Glatiramer acetate**		**IFN beta-1a SC**		**Natalizumab**		**Total**
All DMD users—n (%)^a^	1,827 (22.4)		902 (11.1)		3,261 (40.0)		1,725 (21.1)		800 (9.8)		8,145
DMD monotherapy users—n (%)	1,718 (22.0)		842 (10.8)		3,029 (38.8)		1,544 (19.8)		672 (8.6)		7,805
**Characteristics of DMD monotherapy users in 2011**											
	**IFN beta-1a IM**		**IFN beta-1b**		**Glatiramer acetate**		**IFN beta-1a SC**		**Natalizumab**		***P*** **value**
Demographics											
Gender n (%)^b,c^											0.002
Female	1,313 (76.4)		622 (73.9)		2,354 (77.7)		1,122 (72.7)		501 (74.6)		
Male	404 (23.5)		220 (26.1)		675 (22.3)		422 (27.3)		171 (25.4)		
Mean [SD] age^b^	49.6 [10.1]		47.5 [11.1]		47.3 [11.0]		45.2 [10.5]		45.6 [10.1]		< 0.001
Mean [SD] DMD claims^b,d^	9.21 [4.34]		9.09 [3.91]		5.95 [4.14]		9.03 [4.10]		8.92 [3.98]		< 0.001
Mean [SD] days supply^b,d^	274.6 [93.4]		265.3 [95.5]		186.3 [122.3]		260.9 [105.2]		241.3 [107.8]		< 0.001
Mean [SD] days until DMD start^b,d^	45.80 [67.49]		41.57 [62.24]		76.43 [93.28]		54.17 [77.80]		49.05 [74.87]		< 0.001
Disease sequelae^b^											
Abnormality of gait	104 (6.1)		80 (9.5)		247 (8.2)		133 (8.6)		107 (15.9)		< 0.001
Ataxia	20 (1.2)		26 (3.1)		68 (2.2)		37 (2.4)		29 (4.3)		< 0.001
Burning, numbness, tingling sensations	160 (9.3)		80 (9.5)		305 (10.1)		174 (11.3)		94 (14.0)		0.008
Convulsions	0 (0.0)		0 (0.0)		0 (0.0)		0 (0.0)		0 (0.0)		NA
Depression	125 (7.3)		74 (8.8)		279 (9.2)		143 (9.3)		90 (13.4)		< 0.001
Fecal incontinence	0 (0.0)		0 (0.0)		0 (0.0)		0 (0.0)		0 (0.0)		NA
Fibromyalgia/myalgia and myositis	26 (1.5)		15 (1.8)		84 (2.8)		27 (1.7)		24 (3.6)		0.003
Malaise and fatigue	209 (12.2)		137 (16.3)		493 (16.3)		261 (16.9)		187 (27.8)		< 0.001
Optic neuritis	2 (0.1)		2 (0.2)		4 (0.1)		2 (0.1)		2 (0.3)		0.809
Spasms	33 (1.9)		23 (2.7)		73 (2.4)		39 (2.5)		37 (5.5)		< 0.001
Trigeminal neuralgia	14 (0.8)		5 (0.6)		30 (1.0)		15 (1.0)		9 (1.3)		0.621
Urinary incontinence	31 (0.2)		21 (0.2)		61 (2.0)		23 (1.5)		29 (4.3)		< 0.001
Voice disturbance	4 (0.2)		0 (0.0)		6 (0.2)		1 (0.1)		2 (0.3)		0.449
**MS charge components**^**b**^	**Mean [SD] ($)**	**% of Total**	**Mean [SD] ($)**	**% of Total**	**Mean [SD] ($)**	**% of Total**	**Mean [SD] ($)**	**% of Total**	**Mean [SD] ($)**	**% of Total**	***P*** **value**
Inpatient^e^	2,061 [12,706]	4.5	3,093 [15,185]	6.4	3,053 [18,590]	7.9	2,557 [14,226]	5.6	3,610 [17,308]	4.4	0.155
Outpatient^e^	6,485 [18,322]	14.2	7,931 [27,432]	16.3	7,736 [19,461]	20.1	7,540 [22,866]	16.4	23,702 [26,046]	29.0	< 0.001
Emergency room^e^	15 [323]	0.0	7 [97]	0.0	27 [374]	0.1	28 [364]	0.1	60 [651]	0.1	0.059
DMD	37,209 [13,042]	81.3	37,498 [14,007]	77.3	27,694 [18,055]	71.9	35,803 [14,766]	78.0	54,255 [34,102]	66.5	< 0.001
Total	45,770[25,674]	100.0	48,529 [33,932]	100.0	38,509 [32,570]	100.0	45,928 [30,434]	100.0	81,627 [42,528]	100.0	< 0.001

^a^Counts (%) sum to more than the total DMD user cohort size (100%) because patients could be treated with more than 1 DMD during the year.

^b^Calculated for patients using a single DMD during 2011 (n = 7,805).

^c^Table and chi-square test exclude 1 patient treated with IFN beta-1a with missing gender information.

^d^Includes both medical and pharmacy claims. Days until DMD start represents the number of days from January 1, 2011, until the date of the first DMD claim. For medical claims, days supply was estimated based on the expected dosing schedule for each DMD (count of injections × interval based on dosing schedule).

^e^Charges for medical claims with an MS diagnosis (ICD-9-CM = 340.xx in any diagnosis field or a DRG code for MS).

DMD = disease-modifying drug; IFN = interferon; IM = intramuscular; MS = multiple sclerosis; SC = subcutaneous; SD = standard deviation.

Among monotherapy users, the most notable by-group differences were higher rates of nearly all disease sequelae for natalizumab-treated patients (Table 4). For example, the rate of malaise/fatigue was 28% in the natalizumab monotherapy group compared with 12%-17% for the other DMDs, and abnormality of gait was experienced by 16% in the natalizumab group compared with 6%-10% in the other groups. Natalizumab-treated patients also experienced higher rates of ataxia, paresthesia, depression, fibromyalgia/myositis, spasms, and urinary incontinence. Patients treated with natalizumab monotherapy had the highest mean total MS charges ($81,627), followed by patients treated with IFN beta-1b ($48,529), IFN beta-1a IM and SC ($45,770-$45,928), and glatiramer acetate ($38,509; Table 4). Service categories with significant by-group differences included outpatient (mean $23,702 for natalizumab versus $6,485-$7,931 for the remaining DMDs) and DMD ($54,255 for natalizumab versus $27,694-$37,498 for the remaining DMDs). DMDs accounted for 75% of total MS-related charges overall, ranging from a low of 67% for natalizumab to a high of 81% for IFN beta-1a IM.

In the sample overall from 2006 to 2011, total mean MS charges per patient after adjustment for medical cost inflation increased by 60%, from $16,614 to $26,520 (Table 5). Medical charges rose at a lower rate (33%) than did DMD charges (96%). Expressed as a proportion of the total, medical charges declined from 57% in 2006 to 47% in 2011.

**Table 5:** Comparison of MS charges for 2006 and 2011

**Study year and cohort**	**Service component**		
	**Medical**	**DMD**	**Total**
2006 (n = 15,399)			
Charges ($)	7,966	5,989	13,955
Adjusted charges ($)^a^	9,484	7,130	16,614
Percentage of total charges	57.1%	42.9%	
2011 (n = 15,902)			
Charges ($)	12,567	13,953	26,520
Percentage of total charges	47.4%	52.6%	
Percentage change from adjusted 2006 to 2011	32.5%	95.7%	59.6%

^a^Adjusted for the medical component of the Consumer Price Index.

DMD = disease-modifying drug; MS = multiple sclerosis.

## Discussion

The present study sought to replicate and update a previously published, comprehensive assessment of MS-related health care costs using a retrospective analysis of a large database of commercially insured patients. The study’s primary aim was to provide current data, described in a 2013 comprehensive economic review of MS treatment cost-effectiveness as “sorely needed” because of “advances during the last decade in the care and treatment of MS” [26]. Because pharmacoeconomic models constitute the vast bulk of the literature on MS treatment cost-effectiveness (32 of the 37 studies included in the 2013 review), results of descriptive cost analyses can be, and often are, used as model input values that should be regularly updated as new information becomes available [27].

The study sample was drawn from a large, frequently used, national database of insurance claims and was demographically similar to that of Prescott et al., with a mean age of 47.6 years and percentage female of 76%, compared with 47.1 years and 77% female in the Prescott et al. sample. Results of the present study indicated that total inflation-adjusted MS health care charges increased by 60% from 2006 to 2011 and pointed to a number of important cost drivers.

Foremost, like Prescott et al., we found that common disease sequelae are associated with substantially higher charges for MS-related health care [10]. In both studies, optic neuritis was associated with a charge of more than 200% above average but was rare, affecting 0.4% of the Prescott et al. sample and 0.2% of the present study sample. Abnormality of gait was experienced by 7.4%-7.5% of the patients in both samples; it was associated with charges of 62% and 82% above average in the Prescott et al. study and the present study, respectively.

Malaise and fatigue, depression, and paresthesia were the highest-prevalence disease sequelae in both studies [10]. However, in the present study compared with the study by Prescott et al., these conditions were lower in prevalence (8%-14% vs. 17%-22%, respectively) and associated with greater charge increases (27%-62% above average in the present study vs. no significant difference for paresthesia and charge increases of 8%-13% for depression and malaise/fatigue in the study by Prescott et al.). These variations may be partly due to methodological differences. Specifically, the present study used diagnosis and DRG codes to identify disease sequelae and to calculate MS-related charges, whereas the study by Prescott et al. used the ETG methodology. However, it is possible that changes in MS treatment practices, including the promulgation of several guidelines since 2004, have resulted in changes in the reporting, prevalence, and/or cost of disease sequelae.

The present study found an inverted U-shaped distribution of charges by age, peaking at the ages of 36 to 55 years and dropping among patients aged 65 years or older. This result is similar to that obtained by Prescott et al., although as expected, charges were lower in 2004 than in 2011. Prescott et al. found mean 1-year MS-related charges of $4,464 for those younger than 18 years, $13,130-$13,525 for those aged 36 to 55 years, and $9,677 for those aged 65 years or older.

Unlike Prescott et al., we did not find a decline in medical charges when we examined change over time, although we did find that medical charges increased at a lower rate of growth than did DMD charges in the sample overall, including both DMD users and patients not treated with DMDs. Nonetheless, among DMD monotherapy users in 2011, DMD charges were responsible for 75% of treatment costs, similar to the proportion reported by Prescott et al. (73%) for patients treated with DMD monotherapy in 2004 [10].

The increase in DMD charges from 2006 to 2011 in the sample overall may be attributable to an increased number of available DMDs, as well as price inflation among existing DMDs [28]. A surprising finding of the present study is that the proportion of continuously enrolled patients with at least 1 DMD claim remained constant at about 53%-54% from 2006 to 2011. Also surprising is that the DMD use rate estimated in the present study is less than the 58% reported by Prescott et al. for patients treated in 2004 [10]. It is possible that physicians have either maintained or slightly declined their rate of adoption of DMDs. It is also possible that the diagnosed population has changed over time to include a greater proportion of patients with forms of MS not FDA-approved for treatment with DMDs, such as nonrelapsing MS. ICD-9-CM coding does not distinguish between types of MS and therefore cannot be used to provide an answer to that question.

The addition of natalizumab to the treatment armamentarium in 2006 likely contributed to higher charges for both pharmacy and medical services in the present study. In the analysis of the DMD monotherapy cohort, patients treated with natalizumab incurred the highest MS charges, especially in the outpatient service category. This difference may be partly due to higher rates of disease sequelae associated with greater disease severity, because the Risk Evaluation and Mitigation Strategy (REMS) program under which natalizumab is made available calls for its use only as a second-line drug [24,29,30]. Greater resource utilization for patients treated with natalizumab may also result from REMS program requirements for a schedule of outpatient monitoring and magnetic resonance imaging scan prior to the start of treatment [24,29,30].

### Limitations

The foremost limitations of the present study arise from methodological uncertainty in replicating a study based on a proprietary methodology. Patients in the analysis by Prescott et al. were required to have both a diagnosis of MS and an ETG indicating central nervous system inflammation, and ETG codes were used to capture MS-related charges [10]. Additionally, the database used by Prescott et al. included publicly funded health plans (i.e., Medicare, Medicaid), whereas the current study database was limited to commercially insured patients. Despite these limitations, the age distributions of the samples were similar, and both studies identified similar lists of disease sequelae as cost drivers.

Second, the present study, like that of Prescott et al., was limited to direct health care charges, although indirect costs due to MS are substantial and increase at higher disease severity levels [9]. Additionally, the study’s results are based on U.S. patients and may not generalize to other countries; a systematic review by Naci et al. (2010) found that “cost drivers varied across geographies likely due to the significant differences in the availability of services and resource use patterns across countries” [9]. Finally, because all study patients were continuously enrolled, results cannot be used to draw conclusions about mortality related to MS.

Third, the present study’s finding of lower claims counts and days supply for glatiramer acetate than for the other DMDs among monotherapy users was related to a later start date in calendar year 2011—averages of 76 days (March 18) versus 42 to 54 days (February 12 to February 24), respectively. This finding, which is not consistent with the results of Prescott et al., may be due to plan formulary or benefit design requirements that are not visible within the study database. However, there is no reason to believe that it had a substantial effect on key study results. For example, excluding patients treated with glatiramer acetate, the proportion of total MS cost attributable to DMDs is 76%, similar to the 75% for DMD monotherapy-treated patients overall.

Fourth, although DMD monotherapy users in 2006 might have provided a better point of comparison than the sample of Prescott et al. for the analysis of change in DMD proportional cost over time, use of this subsample was not possible for the 2006 cohort because of difficulties in obtaining detailed cost data. The analysis of 2006 was limited to a secondary objective of the present study.

Fifth, because of its 12-month follow-up time, the present study did not meet the need for longitudinal cohort data for patients with MS [26]. To provide information about the potential cost implications of natural disease history changes due to DMD use, cohort studies with a longer follow-up time are an important area for future research.

## Conclusion

Over a 5-year period from 2006 to 2011, total inflation-adjusted, MS-related charges increased by 60%. MS-related costs among patients treated with different DMDs ranged from 1-year means of $38,509 to $81,627. For DMD monotherapy users, 75% of total MS-related health care cost in 2011 was for DMDs, similar to the percentage previously reported for patients treated in 2004. Sequalae of MS contributed significantly to the total cost of care.

## Competing interests

This work was sponsored by Teva Pharmaceuticals, which manufactures glatiramer acetate. Carroll is a former employee and former stockholder of Teva. Fairman and Lage are consultants to Teva and were compensated by Teva for their work on this study and on other studies. Teva funded the article processing charge for the manuscript. The authors have no additional financial competing interests and no nonfinancial competing interests.

## Authors’ contributions

CC had primary responsibility for study concept and design. ML performed data analyses. KF drafted and edited the manuscript. All authors made substantial contributions to concept/design and data interpretation, participated in critical review and revision of the manuscript, and take public responsibility for its content. All authors read and approved the final manuscript.

## Pre-publication history

The pre-publication history for this paper can be accessed here:

http://www.biomedcentral.com/1472-6963/14/286/prepub
